# Supplementary material for: Identification of photoperiod-induced specific miRNAs in the adrenal glands of Sunite sheep (Ovis aries)
Source: Front Vet Sci. 2022 Jul 22;9:888207. doi: 10.3389/fvets.2022.888207 (PMC9354845; doi:10.3389/fvets.2022.888207)
Supplement: Supplementary file 1 [file Data_Sheet_1.ZIP › Supplementary file/miRDeep2 file.zip!/miRDeep2 file/Know_Structure/SP42A1/oar-mir-23a.pdf]

miRBase precursor : oar-mir-23a
Total read count : 44862
oar-miR-23a read count : 22471
oar-miR-23b read count : 22388
remaining reads : 3

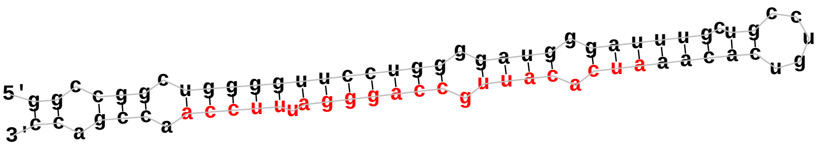

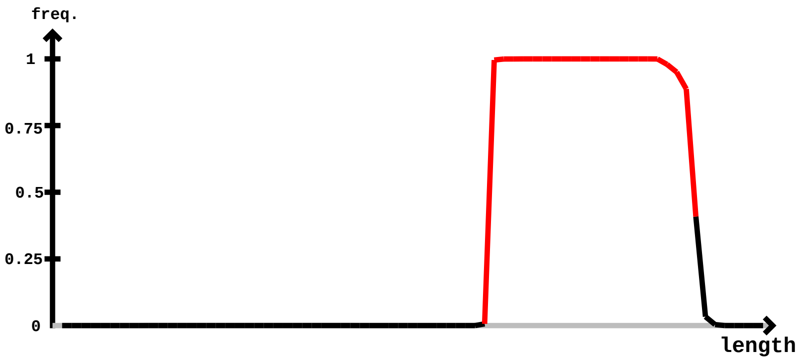


```
                                            oar-miR-23a
                                            oar-miR-23b
5'- ggccggcugggguuccuggggaugggauuugcugccugucacaaaucacauugccagggauuuccaaccgacc   -3'    exp
    ((.(((.((((((((((((.((((.((((((.((.....)))))))).)))).))))))).))))).))).))   reads      mm         sample
    ........gggguuccuggggaugggauu............................................   1          0          seq
    ........gggguuccuggggaugggauuu...........................................   2          0          seq
    ...........................................aaucacauugccagggauu...........   2          0          seq
    ...........................................aaucacauugccagggauuu..........   4          0          seq
    ...........................................aaucacauugccagggauuuc.........   9          0          seq
    ...........................................aaucacauugccagggauuucc........   83         0          seq
    ...........................................aaucacauugccagggauuucca.......   43         0          seq
    ...........................................aaucacauugccagggauuuccaa......   1          0          seq
    ............................................aucacauugccagggau............   6          0          seq
    ............................................aucacauugccagggaC............   1          1          seq
    ............................................aucacauugccagggauu...........   451        0          seq
    ............................................aucacauugcUagggauu...........   1          1          seq
    ............................................aucacauugccUgggauu...........   1          1          seq
    ............................................aucacauugccagggauuu..........   640        0          seq
    ............................................aucacauugccagggauuuc.........   1408       0          seq
    ............................................aucacauugccagggauuucc........   10648      0          seq
    ............................................aucacauugccagggauuucca.......   8345       0          seq
    ............................................aucacauugccagggauuuccaa......   659        0          seq
    ............................................aucacauugccagggauuuccaac.....   74         0          seq
    ............................................aucacauugccagggauuuccaacc....   1          0          seq
    .............................................ucacauugccagggauu...........   3          0          seq
    .............................................ucacauugccagggauuu..........   3          0          seq
    .............................................ucacauugccagggauuuc.........   3          0          seq
    .............................................ucacauugccagggauuucc........   27         0          seq
    .............................................ucacauugccagggauuucca.......   36         0          seq
    .............................................ucacauugccagggauuuccaa......   6          0          seq
    .............................................ucacauugccagggauuuccaac.....   7          0          seq
    .............................................ucacauugccagggauuuccaacc....   1          0          seq
    ..............................................cacauugccagggauuucc........   1          0          seq
    ...............................................acauugccagggauuucc........   4          0          seq
    ...............................................acauugccagggauuucca.......   3          0          seq
```
